# Natural Bioactive Compounds Targeting Key Hallmarks of Aging: Functional Food Potential of Spermidine, Fisetin, Berberine, and Urolithin A

**DOI:** 10.3390/nu18152511

**Published:** 2026-08-03

**Authors:** Wojciech Rzeski, Weronika Rzeska

**Affiliations:** 1 Department of Functional Anatomy and Cytobiology, Institute of Biological Sciences, Maria Curie-Skłodowska University, 19 Akademicka Street, 20-033 Lublin, Poland; 2 Doctoral School of Medical Sciences, Medical University of Lublin, 20-093 Lublin, Poland; weronikarzeska@gmail.com

**Keywords:** functional food, bioactive compounds, spermidine, fisetin, berberine, urolithin A, autophagy, senolysis, mitophagy, aging, healthspan

## Abstract

Naturally derived bioactive compounds that modulate aging-associated mechanisms have attracted growing research interest, yet few reviews examine how such compounds might act together. This narrative review examines four mechanistically distinct compounds, namely spermidine, fisetin, berberine, and urolithin A, as emerging functional food ingredients with scientifically supported effects on key hallmarks of human aging. Spermidine, a dietary polyamine from wheat germ and fermented foods, induces autophagy through EP300 inhibition and is associated with reduced all-cause mortality in prospective studies. Fisetin, a flavonoid from strawberries and apples, exerts senolytic activity by selectively eliminating senescent cells via PI3K/AKT and Bcl-2/Bcl-xL inhibition, with emerging clinical evidence. Berberine, an isoquinoline alkaloid from Berberis species, modulates metabolic dysfunction via AMP-activated protein kinase (AMPK) activation and reshapes gut microbiota composition through direct high intraluminal exposure, with the most extensive clinical dataset of the four compounds. Urolithin A, a gut microbiome-derived postbiotic from ellagitannins in pomegranates and nuts, induces mitophagy via PINK1/Parkin and has been evaluated in a growing number of registered human clinical trials. Together, the four compounds primarily target distinct but complementary aging-associated pathways (autophagy, senolysis, metabolic regulation, mitophagy), suggesting rational potential for combined functional food formulations. Limited direct evidence for their combined use and the need for dedicated co-administration studies are discussed, alongside bioavailability, safety, and regulatory considerations.

## 1. Introduction

The global population is aging at an unprecedented rate. According to the World Health Organization, the number of people aged 60 years and older is projected to reach 2.1 billion by 2050 [1]. The associated burden of non-communicable, age-related diseases is among the foremost challenges facing global health systems today. The biological basis of this functional decline was systematized in the landmark hallmarks of aging framework, first proposed by López-Otín et al. in 2013 [2] and subsequently expanded in 2023 to encompass twelve interconnected molecular, cellular, and systemic processes. These include genomic instability, telomere attrition, epigenetic alterations, loss of proteostasis, disabled macroautophagy, deregulated nutrient-sensing, mitochondrial dysfunction, cellular senescence, stem cell exhaustion, altered intercellular communication, chronic inflammation, and dysbiosis [2,3,4]. The framework has been instrumental in identifying tractable molecular targets for interventions capable of delaying or reversing aspects of biological aging.

Dietary interventions are among the most promising translational strategies to emerge from aging biology. Functional foods, broadly defined as foods that provide health benefits beyond basic nutrition through their bioactive constituents [5,6], offer a practical route for translating mechanistic knowledge of aging biology into dietary recommendations and product development. Growing evidence supports specific plant-derived bioactive compounds as modulators of aging mechanisms at concentrations achievable through diet or controlled supplementation [7,8]. The field, however, has largely focused on individual compounds or narrow compound classes, and comprehensive reviews addressing the complementarity of multiple natural bioactive agents across distinct aging pathways remain scarce. This fragmented body of literature makes it difficult to evaluate how mechanistically complementary natural compounds may collectively target multiple hallmarks of aging within a functional food framework.

This narrative review addresses four compounds that collectively cover mechanistically distinct and largely complementary hallmarks of aging: spermidine, a dietary polyamine and potent inducer of autophagy; fisetin, a flavonoid with documented senolytic activity; berberine, an isoquinoline alkaloid with broad metabolic and microbiome-modulating effects; and urolithin A, a gut microbiome-derived postbiotic that selectively induces mitophagy. The four compounds were selected on the basis of four criteria: (i) natural dietary origin or derivation from dietary precursors; (ii) mechanistically characterized effects on defined hallmarks of aging; (iii) availability of at least preliminary human clinical data; and (iv) mechanistic complementarity among the four compounds. This criterion of complementarity explains the exclusion of other well-studied dietary polyphenols such as resveratrol and quercetin. Although both compounds affect aging-related pathways, their principal mechanisms substantially overlap with those already represented here, including AMPK/sirtuin signaling shared with berberine and senescence/inflammation modulation shared with fisetin. By contrast, the four compounds selected for this review were chosen because each primarily engages a distinct hallmark of aging with comparatively limited mechanistic redundancy. Taken together, the four compounds represent a spectrum of dietary bioactive strategies: from direct food constituents (spermidine, fisetin, berberine) to microbiome-mediated postbiotics (urolithin A), collectively addressing autophagy, cellular senescence, metabolic dysregulation, and mitochondrial quality control.

Existing peer-reviewed literature includes individual reviews for each compound and comparative analyses of subsets (notably spermidine and urolithin A as autophagy/mitophagy inducers [7], and spermidine and berberine as caloric restriction mimetics [8]), but no review has previously examined all four compounds within a unified framework centered on functional food applications and aging mechanism complementarity. The present review provides an integrated perspective on the four compounds, with particular attention to their mechanistic complementarity, an area supported by emerging clinical and mechanistic evidence despite the absence of dedicated multi-compound intervention trials. The contribution of this review is therefore integrative rather than mechanistic. The individual molecular pathways described for each compound have largely been established in prior single-compound literature, which we cite throughout; the novel element is the systematic cross-compound synthesis demonstrating their mechanistic non-redundancy across four distinct hallmarks of aging within a single functional food framework.

## 2. Materials and Methods

Literature was identified through systematic searches of PubMed, Scopus, Web of Science, and Google Scholar (through 2025), combining the compound names spermidine, fisetin, berberine, and urolithin A with terms including functional food, hallmarks of aging, autophagy, senescence, senolysis, mitophagy, clinical trial, and bioavailability. Review articles, randomized controlled trials, prospective cohort studies, and mechanistic in vitro and animal studies were included; grey literature and conference abstracts were excluded except where referenced in clinical trial registries. Because the objective was conceptual integration across four mechanistically distinct compounds rather than quantitative pooling of evidence for a single intervention, a formal systematic review protocol (e.g., PRISMA) was not applied; the approach instead follows established conventions for narrative reviews synthesizing heterogeneous evidence streams across compound classes and mechanism types.

## 3. Spermidine: The Dietary Autophagy Inducer

### 3.1. Natural Sources and Dietary Availability

Spermidine is a naturally occurring triamine belonging to the polyamine family, which also includes putrescine and spermine. It occurs in virtually all living organisms and is found in measurable quantities in a wide range of plant-derived foods. Wheat germ constitutes the richest commonly available dietary source, containing up to approximately 337 mg of spermidine per kilogram of dry weight [9,10,11]. Other notable plant sources include soybeans (up to approximately 207 mg/kg), mushrooms, green peas, lentils and other legumes, corn, broccoli, and cauliflower [10]. Among fermented and animal-derived foods, mature cheeses (particularly aged cheddar and brie), natto (fermented soybeans), and fermented sausages contain significant spermidine concentrations due to microbial polyamine biosynthesis during fermentation [9,10].

Systemic spermidine availability is determined by three sources: dietary intake, endogenous biosynthesis from putrescine via spermidine synthase (SRM), and intestinal microbiome synthesis [12]. All three sources decline with age, contributing to the progressive reduction in circulating polyamine levels observed in aging humans, a phenomenon thought to contribute to the age-associated decline in autophagic flux [10,12,13]. Average dietary spermidine intake in Western populations is estimated at approximately 8–12 mg/day, with higher intakes reported in populations adhering to Mediterranean or Japanese dietary patterns rich in legumes, whole grains, and fermented foods [9,10].

### 3.2. Molecular Mechanisms

Spermidine primarily induces autophagy through inhibition of the acetyltransferase EP300 (E1A binding protein p300), a histone acetyltransferase that normally maintains cytoplasmic autophagy-related proteins (ATGs) in an acetylated, inactive state [12,14]. EP300 inhibition leads to hypoacetylation of multiple ATG proteins, including ATG5, ATG7, ATG12, and LC3, derepressing autophagy gene expression and initiating autophagic flux. This mechanism is distinct from the classical mTORC1 inhibition pathway used by rapamycin, suggesting that spermidine acts as an mTOR-independent autophagy inducer [12].

Additional mechanistic targets include activation of AMP-activated protein kinase (AMPK) and upregulation of SIRT1 deacetylase activity, both of which further promote autophagic signaling and mitochondrial quality control [12,15]. Spermidine also modulates the eIF5A (eukaryotic translation initiation factor 5A) hypusination pathway, supporting mitochondrial respiratory complex synthesis and translation fidelity, a mechanism particularly relevant to the maintenance of mitochondrial function in aging tissues [16,17]. At the inflammatory level, spermidine suppresses NLRP3 inflammasome activation and NF-κB-mediated transcription, thereby reducing the senescence-associated secretory phenotype (SASP) and systemic low-grade inflammation characteristic of aging [12,13].

Together, these mechanisms position spermidine as a pleiotropic caloric restriction mimetic. It recapitulates many of the cellular adaptations induced by dietary restriction, including autophagy induction, mitochondrial optimization, and anti-inflammatory signaling, without requiring caloric restriction itself [8,13,18].

### 3.3. Preclinical Evidence

Spermidine administration extends lifespan in multiple model organisms including *Saccharomyces cerevisiae*, *Caenorhabditis elegans*, *Drosophila melanogaster*, and mice through autophagy-dependent mechanisms, with genetic disruption of autophagy (*atg* gene knockouts) abolishing the longevity-extending effects [19,20].

In aged mice, oral spermidine supplementation reduced cardiac hypertrophy, preserved diastolic function, enhanced cardiac autophagy and mitophagy, and improved mitochondrial respiration [19]. Notably, cardioprotective effects were absent in cardiomyocyte-specific *Atg5*-knockout mice, providing direct in vivo evidence for the autophagy-dependency of spermidine’s cardiovascular benefits [19]. In hypertensive rat models, spermidine feeding reduced systolic blood pressure, increased titin phosphorylation, and prevented progression to heart failure [19].

Neuroprotective effects have been documented in multiple rodent models of neurodegeneration. Spermidine supplementation reduced tau hyperphosphorylation, attenuated amyloid-β accumulation, and preserved spatial memory in murine Alzheimer’s disease models [21]. Additional preclinical evidence supports immunomodulatory benefits, including enhancement of T cell-mediated anticancer immunosurveillance and restoration of age-related immune decline [22,23].

### 3.4. Human Clinical Evidence

Currently, the strongest epidemiological evidence for dietary spermidine comes from the prospective Bruneck Study cohort (*n* = 829, aged 45–84 years, 20-year follow-up), which demonstrated that higher dietary spermidine intake was significantly and independently associated with reduced all-cause mortality [24]. All-cause mortality rates decreased across increasing thirds of spermidine intake from 40.5 to 23.7 and 15.1 deaths per 1000 person-years [24], corresponding to an adjusted reduction in 20-year cumulative mortality of approximately 10 percentage points between the lowest and highest intake tertiles. These associations were robust against unmeasured confounding and were independently replicated in the Salzburg Atherosclerosis Prevention Program cohort [24].

Randomized controlled trial evidence has focused primarily on cognitive outcomes. The SmartAge phase II trial established the safety and tolerability of a wheat germ-derived spermidine extract (1.2 mg/day) administered for 3 months in older adults with subjective cognitive decline (*n* = 30), with no adverse events attributable to supplementation and preliminary improvements in memory performance in the treatment group [25]. The subsequent 12-month SmartAge phase IIb trial (*n* = 100) showed that spermidine supplementation (0.9 mg/day) did not significantly improve mnemonic discrimination capacity or modulate peripheral inflammatory and autophagy-related biomarkers compared with placebo [26]. The relatively low doses used in the SmartAge trials (0.9–1.2 mg/day, compared with an estimated habitual dietary intake of 8–12 mg/day in Western populations; Section 3.1) likely reflect the conservative dose-escalation strategy typically adopted in early-phase studies involving older adults, rather than an attempt to match or exceed habitual dietary exposure. Whether supplemental doses closer to or exceeding typical dietary intake would produce different clinical outcomes remains untested. Several limitations of the current clinical evidence on spermidine merit acknowledgment. The two completed SmartAge trials were relatively small (*n* = 30 and *n* = 100), single-center studies conducted in cognitively at-risk rather than general aging populations, limiting the generalizability of their findings. In addition, long-term safety data beyond 12 months and evidence regarding hard clinical endpoints, such as cognitive decline and cardiovascular events, remain unavailable. The Bruneck Study, while methodologically robust, is observational and therefore remains susceptible to residual confounding by dietary patterns and lifestyle factors.

The ongoing POLYCAD trial (Denmark, NCT06186102) is evaluating spermidine supplementation in patients with coronary artery disease over 48 weeks, with primary endpoints including cardiac function and biomarkers of autophagy and inflammation, representing the first dedicated cardiovascular RCT of dietary spermidine in humans [27].

### 3.5. Potential in Functional Food Formulations

Spermidine’s established safety profile, measurable presence in common foods, and dose-dependent biological effects make it a tractable candidate for functional food development. Key strategies include: enrichment of wheat germ content in cereal-based products; incorporation of natto or natto extracts into fermented food formulations; legume-enriched functional beverages; and standardized wheat germ extract supplements for populations with low dietary intake [9,10].

A practical constraint is that conventionally processed foods may contain reduced spermidine concentrations due to heat-induced degradation, underscoring the importance of processing method optimization. The age-related decline in both dietary intake adherence and endogenous synthesis further strengthens the public health rationale for fortified functional food products targeting older adults [12].

## 4. Fisetin: The Senolytic Flavonoid

### 4.1. Natural Sources and Dietary Availability

Fisetin (3,3′,4′,7-tetrahydroxyflavone) is a naturally occurring flavonol belonging to the polyphenolic flavonoid class. It is present in a range of commonly consumed fruits and vegetables, with strawberries representing the richest dietary source at approximately 160 mg/kg fresh weight [28]. Other quantitatively relevant sources include persimmons (~10.5 mg/kg), apples (~26 mg/kg), grapes (~4 mg/kg), kiwi fruit, peaches, cucumbers, and onions [28]. The flavonol is localized predominantly in the outer layers of fruit and in vegetable skins, rendering it susceptible to losses during peeling and conventional processing.

Bioavailability from dietary sources is substantially limited by fisetin’s poor aqueous solubility, rapid phase II glucuronidation and sulfation in intestinal epithelial cells, and extensive first-pass hepatic metabolism [29]. Plasma concentrations following ingestion of fisetin-containing foods are typically in the low nanomolar range, considerably below the micromolar concentrations required for senolytic activity in preclinical models. This pharmacokinetic constraint is the principal translational challenge for dietary fisetin and has motivated the development of advanced delivery systems and intermittent high-dose supplementation strategies.

### 4.2. Molecular Mechanisms

Fisetin exerts senolytic activity primarily through inhibition of the PI3K/AKT pro-survival signaling axis, which is constitutively activated in senescent cells and renders them resistant to apoptosis [29,30]. AKT inhibition leads to reduced phosphorylation and nuclear exclusion of FOXO transcription factors, impairs NF-κB-mediated pro-survival gene expression, and lowers the apoptotic threshold selectively in senescent cells. Complementary senolytic targets include direct inhibition of anti-apoptotic Bcl-2 family proteins, notably Bcl-2 and Bcl-xL, reducing the mitochondrial apoptotic threshold in cells dependent on these proteins for survival [29,31].

In addition to its senolytic activity, fisetin exerts senomorphic effects through suppression of the NF-κB transcription factor and downstream SASP components, including interleukin-6 (IL-6), interleukin-8 (IL-8), and matrix metalloproteinases (MMP-1, MMP-3) [32]. Additional mechanisms include activation of the Nrf2 antioxidant response pathway, modulation of the mTOR/AMPK energy-sensing axis, inhibition of NLRP3 inflammasome assembly, and activation of SIRT1-dependent deacetylase activity. Together, these support fisetin’s dual character as both a senolytic (cell-eliminating) and senomorphic (SASP-suppressing) compound, depending on dose and cellular context [32,33].

Neuroprotective effects include inhibition of tau hyperphosphorylation through GSK-3β modulation, reduction of amyloid-β aggregation, and activation of ERK1/2 survival signaling in neurons, mechanisms relevant to age-related cognitive decline and neurodegeneration [33,34,35].

### 4.3. Preclinical Evidence

The landmark preclinical study by Yousefzadeh et al. (2018) screened a panel of ten flavonoid polyphenols for senolytic activity using senescent murine and human fibroblasts and identified fisetin as the most potent senolytic compound in the panel, selectively eliminating approximately 25–50% of senescent cells in vitro at concentrations of 1–10 µM [29]. In aged mice, continuous dietary administration of fisetin (500 mg/kg diet) beginning at 85 weeks of age significantly extended both median and maximum lifespan. In a separate acute-dosing paradigm, oral gavage of fisetin (100 mg/kg/day for 5 days) in aged mice reduced senescent cell burden in multiple tissues and decreased expression of SASP components. Importantly, these beneficial effects were observed when treatment commenced in aged animals, establishing translational relevance [29].

Murray et al. (2025) demonstrated that intermittent oral fisetin supplementation (1 week on–2 weeks off–1 week on protocol) in old mice significantly improved frailty scores and grip strength compared to age-matched controls, accompanied by downregulation of senescence-related transcripts (*Cdkn1a*, *Ddit4*) in skeletal muscle [30]. Of particular relevance, the magnitude of improvement with fisetin was comparable to that achieved by genetic clearance of p16^+^ senescent cells and by the synthetic senolytic ABT-263, providing proof-of-concept that a natural dietary compound can achieve senolytic efficacy equivalent to genetic or pharmacological approaches in vivo [30].

Fisetin’s antioxidant, anti-inflammatory, and senolytic activities have also been highlighted as potentially relevant to vascular aging, with proposed benefits including protection of endothelial function and attenuation of SASP-driven vascular inflammation, though dedicated vascular aging trials remain to be conducted [36].

### 4.4. Human Clinical Evidence

Clinical evidence supporting fisetin as a senolytic remains at an early, albeit rapidly evolving, stage. It is essential to distinguish clearly between the strong preclinical and mechanistic evidence base and the substantially more limited clinical validation. Unlike berberine, which has been evaluated in a meta-analysis pooling 46 randomized controlled trials with consistent evidence across glycemic, lipid, and inflammatory endpoints (see Section 5.4), fisetin has no completed phase III trials and only a small number of early-phase human studies with modest sample sizes. The available human data support safety and tolerability but do not yet establish clinical efficacy for senolytic endpoints in adequately powered, placebo-controlled trials. The most comprehensive review of available phase I/II trial data, by Tavenier et al. (2024) with co-authorship from the Mayo Clinic senolytic research group, concluded that fisetin induces apoptosis in senescent cells in several but not all cell types in vitro, reduces senescent cell burden in animal models, and has a favorable safety profile in early human studies, while noting that pharmacokinetic limitations and the absence of validated circulating biomarkers of senolysis remain key translational barriers [31].

Among ongoing and recently completed human trials, the STOP-Sepsis trial (NCT05758246) is evaluating fisetin’s ability to reduce senescent immune cell accumulation and prevent clinical deterioration in elderly patients with sepsis in a multicenter, randomized, double-blind phase II study with adaptive allocation. To date, it represents the most rigorous clinical assessment of fisetin’s senolytic activity [37]. Additional registered trials are investigating fisetin in peripheral arterial disease (NCT06399809), frailty in older adults, and post-COVID condition [29].

Safety data across available human studies indicate that fisetin is well tolerated at doses ranging from 100 mg to 1000 mg/day, with no serious adverse events attributable to supplementation reported in published trials. Gastrointestinal effects have been noted at high doses in a subset of participants [29]. Overall, no convincing evidence currently exists that fisetin produces clinically meaningful senolytic effects in humans; the available data establish safety and biological plausibility but fall well short of demonstrating clinical efficacy.

### 4.5. Bioavailability Challenges and Functional Food Strategies

The primary translational limitation of fisetin is its poor oral bioavailability, driven by low aqueous solubility (log P ≈ 2.9–3.2), rapid glucuronidation in the gut wall and liver, and short elimination half-lives [29,30]. Several formulation strategies are under active investigation to address this constraint. Complexation of fisetin with galactomannan fiber (fenugreek-derived) has been reported to increase bioavailability by more than 25-fold compared to unformulated fisetin in a randomized crossover study in healthy volunteers [38]. Additional approaches include liposomal encapsulation, polymeric nanoparticle delivery, self-emulsifying drug delivery systems (SEDDSs), and co-crystallization with absorption enhancers [36].

From a functional food perspective, the intermittent dosing paradigm shown to be efficacious in preclinical models may align favorably with food product design. Periodic consumption of high-fisetin foods or supplemented products, rather than daily continuous intake, could be sufficient to achieve senolytic effects, provided bioavailability limitations are adequately addressed. Strawberry-based functional food formulations, fisetin-enriched extracts added to beverages, and standardized capsule supplements represent the primary delivery vehicles under consideration [29,32].

## 5. Berberine: The Metabolic and Microbiome Modulator

### 5.1. Natural Sources and Functional Food Context

Berberine is an isoquinoline alkaloid present in the roots, rhizomes, bark, and stems of numerous plant species across multiple families. Quantitatively significant plant sources include *Berberis vulgaris* (barberry), *Berberis aristata* (Indian barberry), *Coptis chinensis* (Chinese goldthread), *Hydrastis canadensis* (goldenseal), and *Phellodendron amurense* (Amur cork tree), with berberine concentrations in roots and bark ranging from less than 1% to more than 8% dry weight depending on species, plant part, and growing conditions [39].

In contrast to spermidine and fisetin, berberine is not a constituent of commonly consumed Western foods at nutritionally relevant concentrations. Its dietary relevance derives principally from its long history of use in traditional Chinese medicine and Ayurvedic practice, as well as from its current status as a regulated dietary supplement and bioactive ingredient used in functional food formulations in multiple jurisdictions. Berberine-containing products include standardized plant extracts, encapsulated supplements, and functional beverages. The compound’s well-documented metabolic effects and favorable safety profile relative to pharmaceutical alternatives have driven substantial consumer interest, making it one of the most commercially significant botanical supplements globally.

Berberine occupies a unique position at the interface between functional foods, nutraceuticals and botanical medicines.

### 5.2. Molecular Mechanisms

The primary mechanism of berberine action is activation of AMPK through inhibition of mitochondrial respiratory chain complex I, which reduces cellular ATP production, elevates the AMP:ATP ratio, and triggers AMPK phosphorylation at Thr172 [40]. AMPK activation initiates a coordinated metabolic shift from anabolic to catabolic processes: increased glucose transporter GLUT4 translocation and glucose uptake in skeletal muscle, enhanced fatty acid β-oxidation, inhibition of lipogenic gene expression through SREBP-1c downregulation, and induction of autophagic flux through mTORC1 suppression [41,42]. These effects closely parallel the metabolic adaptations induced by caloric restriction and exercise, suggesting that berberine functions as a pharmacomimetic of energy deficit signaling.

Anti-inflammatory mechanisms include suppression of NF-κB nuclear translocation, inhibition of NLRP3 inflammasome assembly [43], reduction of TNF-α and IL-6 secretion, and modulation of macrophage polarization from pro-inflammatory M1 to anti-inflammatory M2 phenotype [41]. Berberine also upregulates SIRT1 deacetylase activity and activates the Nrf2-ARE (antioxidant response element) antioxidant response pathway, both of which contribute to attenuation of oxidative stress-driven cellular aging [40,44].

A distinctive feature of berberine among the four compounds reviewed here is its profound modulatory effect on gut microbiota composition. Berberine’s low oral bioavailability (estimated at 1–5%) results in high intraluminal concentrations that selectively reshape the microbiome: increasing the abundance of beneficial bacteria including *Akkermansia muciniphila*, *Bifidobacterium* spp., and short-chain fatty acid (SCFA)-producing *Lactobacillus* species, while reducing conditionally pathogenic taxa associated with metabolic endotoxemia [43]. This microbiome-mediated dimension creates a systemic anti-inflammatory and metabolic effect that operates independently of, and in addition to, berberine’s direct cellular actions. It also contributes to gut barrier integrity and systemic immune tone.

### 5.3. Preclinical Evidence

In *Caenorhabditis elegans*, berberine extended mean lifespan by approximately 27% through coordinated activation of the DAF-16/FOXO, HSF-1, and SKN-1/NRF2 transcription factors (the canonical longevity-regulatory pathways in this model organism), alongside favorable modulation of lipid and fatty acid metabolism [44]. In rodent models of insulin resistance and type 2 diabetes, berberine consistently improved glucose tolerance, reduced fasting glycemia, lowered plasma triglycerides and LDL cholesterol, and attenuated hepatic steatosis across multiple animal models and experimental paradigms [41].

In naturally aging rats, berberine administration for six months improved cognitive function, reversed insulin resistance in skeletal muscle, and increased mitochondrial biogenesis markers (p-AMPK, SIRT1, PGC-1α, ATP production) in skeletal muscle tissue. These findings provide preclinical evidence for berberine’s capacity to address age-related sarcopenia and cognitive decline through mitochondrial mechanisms [42]. Berberine has additionally been shown to attenuate premature cellular senescence in human fibroblasts exposed to oxidative stress, an effect attributed to SIRT1-mediated deacetylation of p53 and consequent delay of p21-dependent cell cycle arrest [40].

### 5.4. Human Clinical Evidence

Berberine possesses the most extensive clinical evidence base among the four compounds reviewed here. This evidence spans head-to-head pharmaceutical comparisons, large-scale meta-analyses pooling dozens of randomized controlled trials across several thousand patients with type 2 diabetes and related metabolic conditions, and a dedicated microbiome-focused trial, summarized below. The landmark head-to-head comparison by Yin et al. (2008) randomized 36 newly diagnosed type 2 diabetes patients to berberine (500 mg three times daily) or metformin (500 mg three times daily) for 3 months, demonstrating comparable reductions in HbA1c, fasting blood glucose, postprandial blood glucose, and triglycerides in both groups [45]. This was the first direct evidence that a natural alkaloid could achieve glycemic efficacy equivalent to a first-line pharmaceutical agent [45].

Subsequent meta-analyses have consolidated this evidence. A systematic review and meta-analysis of 46 randomized controlled trials in type 2 diabetes patients (Guo et al., 2021) demonstrated that berberine significantly reduced HbA1c (MD = −0.73%; 95% CI: −0.97 to −0.51), fasting plasma glucose (MD = −0.86 mmol/L), 2-h postprandial glucose (MD = −1.26 mmol/L), and HOMA-IR (MD = −0.71) [46]. The same analysis also demonstrated reductions in total cholesterol (MD = −0.64 mmol/L), LDL-C (MD = −0.86 mmol/L), and triglycerides (MD = −0.50 mmol/L), with simultaneous reductions in inflammatory biomarkers C-reactive protein (CRP), IL-6, and TNF-α [46]. A subsequent umbrella meta-analysis of randomized controlled trials (2023/2024) corroborated these findings, confirming consistent glycemic, lipid, and anti-inflammatory efficacy across the accumulated clinical trial literature [47].

The clinical evidence extends beyond metabolic parameters. The PREMOTE trial, a multi-center RCT in approximately 400 newly diagnosed type 2 diabetes patients, demonstrated that berberine reduced HbA1c by approximately 1% at 12 weeks and produced significant, measurable changes in gut microbiota composition and microbial metabolite profiles, directly linking clinical metabolic improvement with microbiome-mediated mechanisms in humans [48].

### 5.5. Bioavailability and Delivery Systems

Berberine’s paradoxically useful pharmacokinetic profile, characterized by low systemic bioavailability of 1–5% due to P-glycoprotein-mediated efflux and extensive first-pass metabolism combined with high intraluminal concentrations, means that its gut microbiome-modulating effects may be favored by conventional oral formulations, while systemic AMPK activation in peripheral tissues requires adequate absorption [40].

To address the latter, advanced delivery strategies include dihydroberberine (BBR-H2), a reduced form with markedly improved intestinal absorption that is re-oxidized to berberine in tissues; phospholipid complexes (berberine-phosphatidylcholine); nanoparticle encapsulation; and solid dispersions in hydrophilic polymers [40]. Berberine-ursodeoxycholic acid salt formulations have been evaluated in recent clinical trials for improved bioavailability and hepatic targeting [49]. The choice of formulation for functional food applications should be informed by whether the primary therapeutic goal is systemic metabolic modulation (favoring enhanced-absorption forms) or microbiome reshaping (where conventional berberine may be sufficient or even preferable).

## 6. Urolithin A: The Dietary Postbiotic and Mitophagy Activator

### 6.1. Ellagitannins as Dietary Precursors and Food Sources

Urolithin A (UA; 3,8-dihydroxy-6H-dibenzo[b,d]pyran-6-one) is not present in food per se but is produced endogenously through the intestinal microbiome’s transformation of ellagitannins and ellagic acid, polyphenols naturally occurring in a range of plant-based foods [50,51,52]. These three terms describe sequential stages of the same pathway rather than interchangeable substances: ellagitannins are the parent dietary polyphenols; ellagic acid is their direct hydrolysis product, released in the small intestine; and urolithin A is the terminal, colon-microbiota-derived metabolite responsible for the bioactivity discussed in this section. Urolithin A is commonly described in the literature as a gut microbiome-derived postbiotic metabolite, reflecting its origin as a product of microbial metabolism rather than a dietary constituent per se; its formal classification under the widely adopted International Scientific Association of Probiotics and Prebiotics (ISAPP) definition of “postbiotic”, namely a preparation of inanimate microorganisms and/or their components that confers a health benefit on the host, remains subject to terminological debate, since a single purified microbial metabolite does not straightforwardly fit a definition centered on microbial preparations. We use “microbiome-derived metabolite” and “postbiotic” interchangeably in this review, consistent with common usage in the field. Principal dietary precursor sources include pomegranate (fruit, juice, and peel), which contains the highest ellagitannin density (principally punicalin and punicalagin), followed by walnuts, pecans, almonds, raspberries, strawberries, blackberries, oak-aged wines, and certain herbal teas [52,53]. Ellagitannins are hydrolyzed to ellagic acid in the gastrointestinal tract, which is subsequently converted by specific gut bacteria to urolithins through a reductive lactonization cascade involving sequential removal of hydroxyl groups.

The principal bacterial taxa responsible for urolithin A biosynthesis are *Gordonibacter urolithinfaciens* and *Ellagibacter isourolithinifaciens*, with contributions from select *Enterocloster* species [52]. This conversion is a multi-step process requiring a specific microbial consortium that is not universally present in the human gut. The pathway proceeds from ellagic acid through urolithin M6 and urolithin C to urolithin A as the terminal and predominant metabolite in efficient converters, with urolithin B (a dehydroxylated form) produced by a subset of individuals [53].

### 6.2. Gut Microbiome Conversion and Inter-Individual Variability

One of the defining characteristics of urolithin A biology is the marked interindividual variability in production capacity from dietary ellagitannin intake [51,52]. Population studies have classified individuals into three urolithin metabotypes: Metabotype A (UA producers), Metabotype B (producers of urolithin B and isoUrolithin A but not UA), and Metabotype 0 (non-producers), regardless of ellagitannin consumption. The original characterization of a Western cohort reported approximately 40% Metabotype A, 10% Metabotype B, and 50% Metabotype 0. However, substantial inter-population variability has since been documented, with Metabotype A prevalence ranging from roughly 25% to 55% and Metabotype 0 from approximately 10% to 50%, depending on ethnicity, geography, and cohort characteristics [51,52]. This classification implies that dietary strategies relying on ellagitannin-rich food consumption will be ineffective for achieving urolithin A exposure in the majority of the population, a fundamental constraint distinguishing UA from the other three compounds reviewed here.

This interindividual variability has driven the development of direct urolithin A supplementation, bypassing microbiome dependency. Unlike dietary ellagitannins, direct supplementation offers consistent systemic exposure independent of individual differences in gut microbiota composition.

### 6.3. Molecular Mechanisms

Urolithin A was identified as the first naturally occurring compound to induce mitophagy (the selective autophagic elimination of damaged or dysfunctional mitochondria) both in vitro and in vivo following oral consumption, by Ryu et al. in 2016 [54]. The primary mechanistic pathway involves stabilization and accumulation of PINK1 (PTEN-induced kinase 1) on the outer mitochondrial membrane of depolarized mitochondria, leading to phosphorylation and activation of the E3 ubiquitin ligase Parkin (PRKN). Parkin-mediated ubiquitination of outer mitochondrial membrane proteins generates recognition signals for autophagy receptors (p62/SQSTM1, NDP52, OPTN), initiating selective engulfment of the damaged mitochondrion by the autophagosome and subsequent lysosomal degradation [55].

### 6.4. Preclinical Evidence

In *C. elegans*, UA extended median lifespan by 45.4% at 50 µM, the most potent longevity effect among the four urolithin metabolites tested, through mitophagy-dependent mechanisms confirmed by genetic ablation of *dct-1* (the nematode BNIP3L ortholog) and the autophagy gene *bec-1* [54]. UA also prevented the age-related accumulation of dysfunctional mitochondria, preserved pharyngeal pumping and mobility in aged worms, and extended healthspan independently of changes in feeding behavior [54].

Rodent studies have demonstrated that UA supplementation improved exercise capacity and skeletal muscle mitochondrial function in a Duchenne muscular dystrophy mouse model [56]. Cardiovascular benefits have been documented in preclinical models, with UA restoring mitochondrial function, reducing cardiac hypertrophy, and reversing cardiac remodeling in models of heart failure with preserved ejection fraction, effects mediated through mitophagy induction in cardiomyocytes [57].

### 6.5. Human Clinical Evidence

Urolithin A has been evaluated in a growing number of registered human clinical trials. A 2024 systematic review identified five completed trials (*n* = 250 total participants) assessing mitochondrial, inflammatory, and muscle-function outcomes, with numerous additional trials registered or ongoing on ClinicalTrials.gov. This is the largest peer-reviewed clinical dataset among naturally occurring mitophagy-inducing compounds [58]. The first-in-human phase I trial by Andreux et al. (2019, *Nature Metabolism*) established a favorable safety and bioavailability profile for UA at single doses of 250–2000 mg and multiple doses of 500 and 1000 mg/day over 4 weeks in healthy sedentary elderly individuals [59]. Both 500 mg and 1000 mg doses significantly modulated plasma acylcarnitine profiles and induced mitochondrial gene expression in skeletal muscle biopsies, providing the first human evidence of target engagement (mitophagy pathway activation) following oral UA consumption [59].

The ATLAS randomized controlled trial (Singh et al., 2022, *Cell Reports Medicine*) enrolled 88 middle-aged adults (NCT03464500) to receive UA (500 or 1000 mg/day) or placebo for 4 months [60]. UA supplementation produced significant improvements in muscle strength (~12% increase in hamstring strength at 500 mg/day), aerobic endurance (peak VO_2_), and 6-min walk test distance, alongside reductions in plasma acylcarnitines and C-reactive protein, consistent with mitochondrial efficiency improvements and anti-inflammatory effects [60].

A parallel trial in older adults (Liu et al., 2022, *JAMA Network Open*; NCT03283462) demonstrated that UA (1000 mg/day for 4 months) significantly improved skeletal muscle endurance in both hand (first dorsal interosseous) and leg (tibialis anterior) muscles compared to placebo, with accompanying improvements in plasma mitochondrial health biomarkers [61]. The most recent RCT, published in *Nature Aging* (2025), demonstrated that UA supplementation (1000 mg/day for 4 weeks) produced significant remodeling of the peripheral immune landscape in healthy middle-aged adults, expanding naive T cell and TSCM populations and reprogramming T cell metabolic profiles toward oxidative phosphorylation, consistent with modulation of immune aging-related phenotypes [62].

### 6.6. Dietary Strategy vs. Direct Supplementation

Given the substantial interindividual variability in microbiome-mediated ellagitannin-to-urolithin A conversion, consumption of ellagitannin-rich foods (pomegranate juice, walnut-enriched products, berry formulations) yields reliable UA exposure only in Metabotype A individuals, a subgroup reported to comprise roughly a quarter to half of most studied populations [51,52]. For the remaining individuals, dietary precursor intake does not reliably translate to measurable UA exposure. This has two implications for functional food design: first, ellagitannin-enriched products may benefit a defined population subgroup that can be identified through urolithin metabotyping; second, direct supplementation with purified UA (as Mitopure or equivalent) delivers consistent, dose-controlled systemic exposure independent of microbiome composition [59]. Hybrid strategies combining prebiotic support for *Gordonibacter* and *Ellagibacter* growth, together with ellagitannin intake, remain promising but understudied.

## 7. Complementary Mechanisms and the Combination Hypothesis

### 7.1. Mechanistic Complementarity Within the Hallmarks of Aging Framework

**Figure 1:**
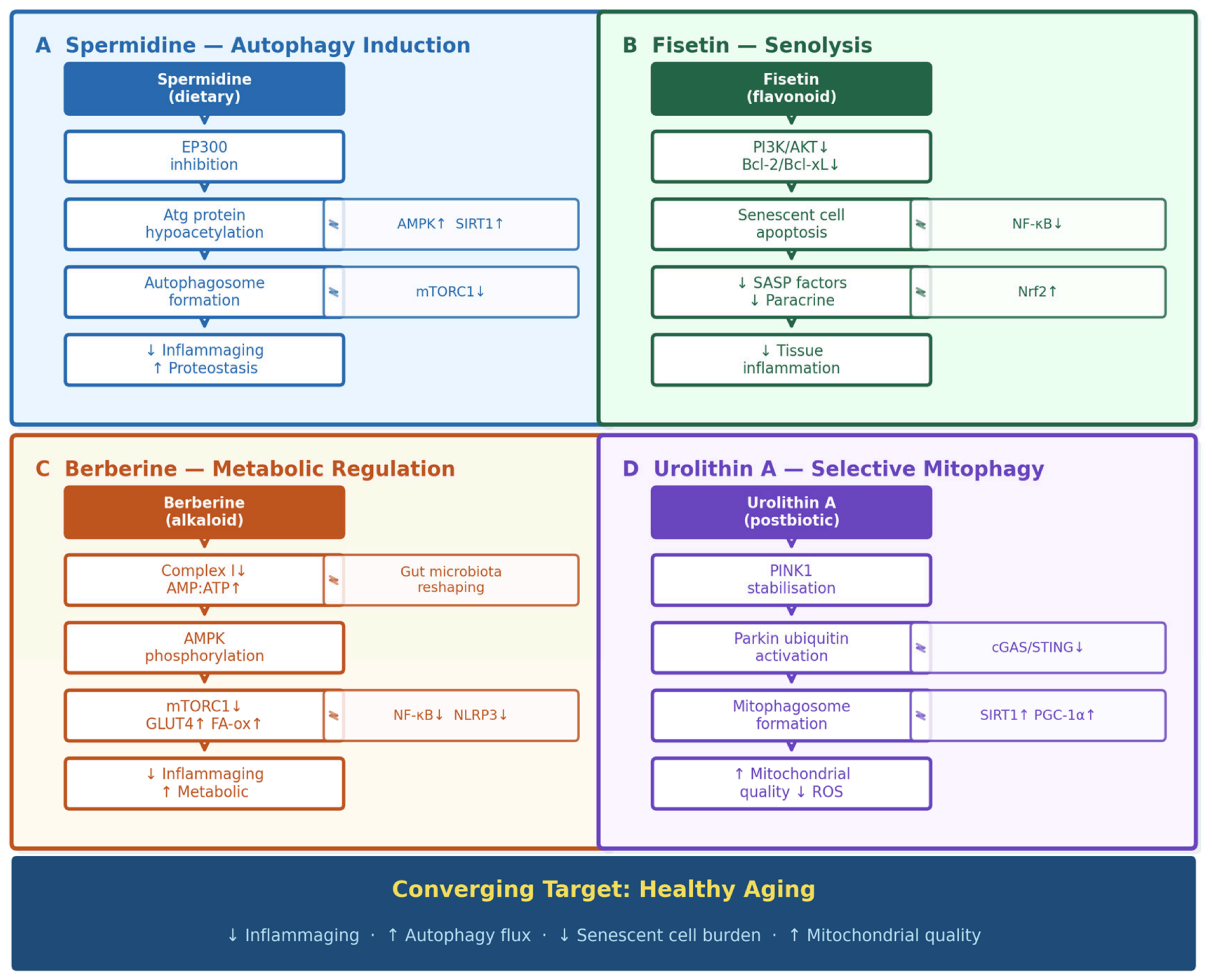
Mechanistic complementarity of the four reviewed bioactive compounds. This is an original, author-generated conceptual schematic. Each panel illustrates the primary signaling cascade for spermidine (**A**), fisetin (**B**), berberine (**C**), and urolithin A (**D**), each summarized from the single-compound evidence cited in the corresponding section of the main text, converging on shared downstream outcomes relevant to healthy aging. Abbreviations are as defined in the main text and the Abbreviations list.

The central premise of this review is that spermidine, fisetin, berberine, and urolithin A target distinct, largely independent mechanisms within the hallmarks of aging framework while converging on common downstream processes, namely mitochondrial health, reduced chronic inflammation, and preservation of cellular homeostasis (Figure 1) [2,3]. This complementarity provides the rationale for considering these compounds as a multi-target strategy for functional food development.

Spermidine primarily induces general (bulk) autophagy through EP300 inhibition, thereby maintaining proteostasis, clearing damaged organelles broadly, and suppressing NLRP3-mediated inflammaging [12,13]. Urolithin A, by contrast, induces selective mitophagy (the targeted elimination of damaged mitochondria specifically) through the PINK1/Parkin pathway [54,55]. These two forms of autophagy are complementary: general autophagy provides global cellular quality control, while mitophagy addresses mitochondrial quality control specifically. Impaired mitophagy, even in the context of preserved general autophagy, results in accumulation of dysfunctional mitochondria, ROS overproduction, and activation of the cGAS/STING pathway and downstream inflammatory signaling [63]. Spermidine and urolithin A thus address complementary aspects of cellular recycling that cannot substitute for each other [63,64,65].

The relationship between autophagy and cellular senescence is further illuminated by a recently proposed threshold model (Bahar et al., 2026), which posits that autophagic flux suppresses senescence initiation below a critical cellular damage threshold, but is reprogrammed to support senescent cell survival once that threshold is exceeded [66]. This model offers a conceptual rationale for combining these compounds: spermidine’s autophagy-enhancing effect may operate most beneficially in pre-senescent cells (preventing senescence initiation), while fisetin’s senolytic activity is required to eliminate established senescent cells that have already crossed this threshold, suggesting that the two compounds act sequentially and non-redundantly on the autophagy–senescence continuum.

Unlike spermidine and urolithin A, fisetin’s senolytic mechanism (selective elimination of PI3K/AKT-dependent and Bcl-2/Bcl-xL-protected senescent cells [29,30]) addresses a distinct cellular population and biological process from the autophagic pathways targeted by spermidine and urolithin A. Senescent cells accumulate dysfunctional mitochondria and produce SASP factors that propagate paracrine senescence to neighboring cells and sustain systemic chronic inflammation. Their elimination through fisetin-mediated senolysis reduces this SASP-driven inflammatory burden and removes a major source of mitochondrial-damaging ROS. Finally, berberine complements these mechanisms by restoring metabolic homeostasis through AMPK activation and gut microbiota remodeling, thereby counteracting nutrient-sensing dysregulation and gut-derived inflammatory signaling that amplify the other hallmarks of aging [41,42,67,68]. This mechanistic complementarity is summarized in Table 1.

### 7.2. Evidence for Combined Use: Pairs and Subsets

Direct evidence for the combined administration of all four compounds simultaneously does not yet exist in the peer-reviewed literature. However, a growing body of indirect and partial evidence supports the mechanistic complementarity of compound subsets and provides a mechanistic rationale for prospective multi-compound trials.

The most direct clinical evidence comes from NCT06990256, a registered randomized, quadruple-blind, placebo-controlled trial (Bonerge/Huazhong University of Science and Technology; *n* = 80, aged 45–70) currently underway, which evaluates single and combined effects of urolithin A (500 mg) and fisetin (500 mg), including a combination arm (UA 300 mg + fisetin 200 mg), on sleep quality and aging biomarkers including DNA methylation age, inflammatory markers, circadian proteins (BMAL1, PER2), and HOMA-IR over 12 weeks [72]. This is, to our knowledge, the only registered trial assessing the combined effect of any two of the four compounds, and its results may help clarify the feasibility of multi-target combination strategies.

At the mechanistic level, a 2025 review comparing spermidine and urolithin A as autophagy/mitophagy inducers concluded that the two compounds operate through distinct but convergent pathways: spermidine through broad EP300-dependent autophagy induction and urolithin A through PINK1/Parkin-specific mitophagy, and proposed that their combination could provide more comprehensive mitochondrial and proteostatic quality control than either compound alone [7]. Similarly, a 2025 analysis comparing spermidine and berberine as caloric restriction mimetics identified complementary rather than redundant mechanisms: spermidine’s primary action through histone deacetylation and autophagy, and berberine’s through AMPK-gut microbiota axes, supporting additive benefits from co-administration [8].

**Figure 2:**
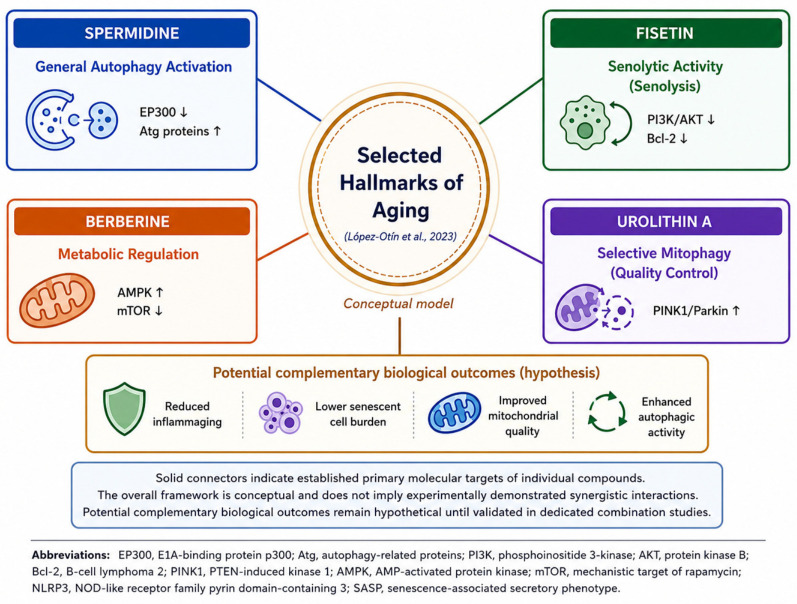
Conceptual illustration of the complementary, non-redundant molecular activities of spermidine, fisetin, berberine, and urolithin A across selected hallmarks of aging. The diagram summarizes evidence discussed in this review and does not imply experimentally demonstrated synergistic effects of combined administration. Potential complementary biological outcomes remain hypothetical until validated in dedicated combination studies [3]. ↑—indicates activation or upregulation; ↓—indicates inhibition or downregulation.

From a conceptual perspective, the four compounds converge on a shared set of hallmarks of aging despite acting through distinct, largely non-overlapping primary molecular targets (Figure 2). Spermidine acts on EP300- and Atg protein-dependent general autophagy; fisetin acts on PI3K/AKT- and Bcl-2-dependent senolysis; berberine acts on AMPK- and mTOR-dependent metabolic regulation; and urolithin A acts on PINK1/Parkin-dependent selective mitophagy. Each compound’s primary mechanism has so far been characterized largely in isolation. Whether their combined use could produce complementary downstream benefits, such as reduced inflammaging, lower senescent cell burden, improved mitochondrial quality, and enhanced autophagic activity, therefore remains a hypothesis rather than an established finding. Although this hypothesis remains untested experimentally and has not yet been directly examined in human co-administration studies, it is consistent with concepts from network medicine suggesting that simultaneous modulation of multiple highly connected regulatory nodes may yield disproportionate biological effects [67,68,72]. Mechanistic complementarity does not necessarily translate into clinical synergy, since pharmacokinetic interactions, disease stage, and individual biological variability may substantially influence treatment outcomes; the proposed multi-target model should therefore be regarded as a scientifically grounded hypothesis awaiting prospective validation rather than as established evidence of combined efficacy. The mechanistic non-redundancy of the four compounds, verified by the fact that genetic or pharmacological ablation of any single pathway (autophagy, senolysis, AMPK signaling, or mitophagy) produces distinct and non-overlapping aging phenotypes in animal models, lends mechanistic support to the hypothesis of additive rather than merely redundant, effects from their co-administration [67,73,74,75].

### 7.3. Practical Considerations for Multi-Compound Functional Food Formulations

Translating this multi-target rationale into functional food or dietary supplement formulations requires attention to several practical parameters. For dosing, the effective dose ranges established in human trials are: spermidine, 0.9–3.3 mg/day (as wheat germ extract); fisetin, 100–1000 mg/day (intermittent dosing preferred); berberine, 900–1500 mg/day (divided doses); and urolithin A, 500–1000 mg/day [25,26,29,30,45,59,60,61]. These doses are achievable in supplement formulations, though food matrix delivery for fisetin and berberine faces bioavailability constraints requiring formulation innovation.

Pharmacokinetic interactions among the four compounds remain unstudied and represent a critical knowledge gap. Berberine is a known inhibitor of CYP3A4 and P-glycoprotein, which could theoretically modulate the absorption and metabolism of co-administered fisetin and urolithin A, an interaction warranting dedicated investigation before multi-compound formulations are advanced to clinical use. Spermidine’s pharmacokinetics as a dietary polyamine are relatively well-characterized and do not suggest significant interaction potential with the other three compounds at dietary doses.

Population heterogeneity in urolithin A production capacity (Metabotypes A, B, and 0) means that ellagitannin-based dietary strategies will be effective only in a subset of individuals. Metabotype A prevalence ranges from approximately 25% to 55% across studied populations, reflecting the substantial inter-population variability in urolithin metabotypes described in Section 6.2 [51,52]. For multi-component functional food formulations targeting broad population coverage, direct urolithin A supplementation (e.g., as Mitopure) rather than reliance on pomegranate or berry ingredients is the more reliable delivery strategy. Alternatively, including microbiome-modulating components (prebiotics, specific fermented food elements) that support *Gordonibacter* and *Ellagibacter* populations could expand the proportion of effective converters, though this approach remains understudied. These considerations point toward two complementary formulation concepts rather than a single universal product: a broad-coverage formula built around direct urolithin A supplementation, suitable across all metabotypes without requiring prior screening; and a diet-integrated formula combining ellagitannin-rich ingredients with *Gordonibacter*/*Ellagibacter*-supporting prebiotics, positioned for Metabotype A-enriched populations or consumers willing to undergo metabotype screening to confirm converter status.

### 7.4. Priority Directions for Future Research

The multi-target hypothesis advanced here, that spermidine, fisetin, berberine, and urolithin A address partially distinct aging mechanisms and may produce complementary or additive benefits when combined, remains to be tested prospectively. Several research priorities should therefore be addressed. First, dedicated co-administration randomized controlled trials should be conducted in defined aging populations (older adults with metabolic syndrome, frailty, or accelerated biological aging), incorporating composite biomarker panels that address multiple aging hallmarks simultaneously. Second, the pharmacokinetics of the four compounds should be characterized during co-administration, with particular attention to CYP3A4/P-glycoprotein-mediated interactions. Third, participants should be stratified by urolithin metabotype and gut microbiome composition to identify subgroups most likely to benefit from dietary versus supplemental urolithin A delivery. Fourth, composite aging biomarker indices, incorporating markers of autophagy, senescence, mitochondrial function, and metabolic health, should be developed and validated as efficacy endpoints. Fifth, preclinical co-administration studies in aged animal models are needed to establish proof-of-concept for multi-target effects before human translation; a concrete design would randomize aged mice to single-compound arms (spermidine, fisetin, berberine, or urolithin A alone), pairwise-combination arms informed by the mechanistic pairings discussed above, a four-compound combination arm, and vehicle control, with a composite readout panel spanning autophagic flux, senescent cell burden, mitochondrial quality, and systemic inflammatory markers. Finally, pharmacogenomic determinants that may influence individual response, including CYP3A4*3 and CYP2D6 poor-metabolizer alleles affecting berberine exposure [76] and urolithin metabotype-determining gut microbiome signatures, warrant exploration where supported by preliminary evidence; the specific contribution of AMPK α2 (PRKAA2) variants to berberine and spermidine efficacy remains preliminary and requires confirmation.

## 8. Safety, Bioavailability, and Regulatory Considerations

### 8.1. Safety Profiles

Spermidine. As a naturally occurring dietary constituent present in all studied mammalian species and consumed in varying quantities throughout human life, spermidine has an inherent basis for safety at dietary concentrations. In a dedicated preclinical GLP-compliant 90-day oral toxicity study in rats, spermidine trihydrochloride (high-purity synthetic form) produced no adverse effects at doses up to 728–829 mg/kg body weight/day, establishing a wide safety margin relative to supplementary doses used in human trials [77]. In human randomized controlled trials at doses of 0.9–3.3 mg/day (as wheat germ extract), no adverse events attributable to spermidine were reported across safety endpoints including vital signs, clinical chemistry, and hematological parameters [25,26]. A theoretical concern is that polyamine supplementation could support the proliferation of pre-existing cancer cells; however, this effect has not been demonstrated in human supplementation trials to date [12].

Fisetin. Available human trial data indicate that fisetin is well tolerated at doses ranging from 100 mg to 1000 mg/day, with no serious adverse events reported in published trials [29]. Gastrointestinal effects (mild nausea, loose stool) have been noted at higher doses in a subset of participants. Fisetin’s low oral bioavailability limits systemic exposure from standard formulations, which may paradoxically reduce the risk of off-target effects while simultaneously constraining efficacy. A theoretical concern pertains to fisetin’s inhibitory activity on CYP1A2 and CYP3A4 at high concentrations, which could interact with co-administered medications metabolized by these enzymes; however, this has not been clinically documented at doses used in human trials [29].

Berberine. Berberine has an extensive safety record from decades of clinical use in Asia, primarily at doses of 900–1500 mg/day in divided doses [48,78]. The most commonly reported adverse effects are gastrointestinal (nausea, constipation, abdominal discomfort), generally mild and transient. Caution is warranted regarding drug interactions: berberine inhibits CYP3A4, CYP2D6, and P-glycoprotein, potentially elevating plasma concentrations of co-administered drugs including statins, warfarin, cyclosporine, and certain antidiabetic agents [78]. Berberine is contraindicated in pregnancy due to preclinical evidence of teratogenicity and neonatal jaundice risk. EFSA initiated a formal safety assessment of berberine in plant preparations for food supplements in 2023 (EFSA-Q-2022-00803). The NDA Panel endorsed a draft opinion in January 2026, and the subsequent public consultation closed in May 2026; finalization of the opinion is expected to significantly influence the compound’s regulatory standing in the European Union [79].

The clinical relevance of berberine’s CYP3A4 and P-gp inhibitory activity deserves particular attention in the context of multi-compound formulations and polypharmacy. Berberine has been shown to increase plasma concentrations of cyclosporine and to inhibit CYP3A4 activity, with direct clinical consequences for co-administered CYP3A4-metabolized drugs including certain statins and warfarin in vulnerable populations [78]. In the specific context of the four compounds reviewed here, berberine co-administration with fisetin is of concern, as fisetin is itself a CYP3A4 substrate and partial inhibitor; their simultaneous administration could result in mutually elevated plasma levels with unpredictable pharmacodynamic consequences. Genetic polymorphisms in CYP3A4 (notably the *3 allele) and CYP2D6 (poor metabolizer phenotype, ~7% in Europeans [76]) would further amplify interindividual variability in berberine exposure and interaction potential. Until dedicated pharmacokinetic interaction studies are conducted, practitioners should exercise caution when combining berberine with CYP3A4-metabolized medications or with fisetin at high doses. As a pragmatic precaution pending such studies, temporal separation of dosing (e.g., berberine with the morning meal and fisetin in the evening) and avoidance of co-administration in individuals concurrently taking narrow-therapeutic-index CYP3A4 substrates (e.g., certain immunosuppressants or anticoagulants) represent reasonable interim risk-mitigation strategies for any multi-compound formulation combining these two compounds.

Urolithin A. Urolithin A has undergone the most rigorous formal safety evaluation of the four compounds reviewed here. The phase I first-in-human trial established a favorable safety and tolerability profile at single doses up to 2000 mg and multiple doses up to 1000 mg/day for 4 weeks [59]. Across the completed and ongoing RCTs identified in the 2024 systematic review and subsequent registry entries, no serious adverse events attributable to urolithin A have been reported, and clinical chemistry, hematology, and vital sign parameters have remained within normal ranges [58,59,60,61,62]. No clinically significant drug interactions have been identified to date.

Across all four compounds, an important gap common to the existing safety literature is the near-total absence of dedicated, prospectively designed drug-interaction studies and of toxicology data extending beyond approximately 90 days in animal models or 12 months in human trials. Current safety conclusions therefore rest on relatively short observation windows and should be interpreted accordingly, particularly for any future multi-compound formulation intended for long-term daily use.

### 8.2. Bioavailability Comparison

The four compounds exhibit markedly different bioavailability profiles, with important implications for functional food design and dosing strategies. Spermidine, as a small, water-soluble polyamine, is absorbed efficiently from the intestinal lumen through polyamine transporters. Its plasma pharmacokinetics are difficult to characterize by conventional means, however, because absorbed spermidine is rapidly interconverted with spermine and taken up by peripheral tissues; plasma concentrations may therefore not directly reflect systemic exposure. Despite this analytical limitation, systemic bioavailability appears sufficient to modulate autophagy in peripheral tissues at dietary doses [12]. Urolithin A, when administered as purified supplement, demonstrates dose-proportional plasma pharmacokinetics with Tmax of 4–6 h and plasma half-life of approximately 20 h, supporting once-daily dosing [59].

Fisetin and berberine both face significant bioavailability constraints. Fisetin’s oral bioavailability is limited by poor aqueous solubility, rapid phase II conjugation, and a short plasma half-life not yet well characterized in humans, resulting in plasma concentrations from standard formulations that may be insufficient for senolytic activity in peripheral tissues [29,30]. Berberine’s systemic oral bioavailability is estimated at 1–5% due to intestinal P-glycoprotein efflux and first-pass hepatic metabolism, though intraluminal concentrations substantially exceed systemic levels and may be therapeutically relevant for gut microbiome modulation independently of systemic absorption [80]. Table 2 summarizes key pharmacokinetic and bioavailability parameters for the four compounds.

### 8.3. Regulatory Status

The four compounds occupy distinct regulatory positions across major jurisdictions, with implications for their use as functional food ingredients (Table 3).

Spermidine is classified as a dietary supplement in the United States and as a food supplement ingredient in the European Union, where it is marketed primarily as wheat germ extract. No novel food authorization is required for wheat germ-derived spermidine, as it is derived from a traditional food source [12]. High-purity synthetic spermidine trihydrochloride is subject to GRAS self-affirmation procedures in the US; at least one submission supporting its safety has been completed [77].

Fisetin is regulated as a dietary supplement in the United States and as a food supplement in the European Union, where it is generally sold as a plant extract (principally from *Rhus succedanea* or *Strawberry*). No novel food authorization is currently required in the EU for naturally sourced fisetin extracts with a history of use, though this may be subject to review as clinical applications advance [29].

Berberine occupies the most complex regulatory position. In the United States, berberine is sold as a dietary supplement. In the European Union, a critical development occurred in April 2025, when the European Commission terminated the novel food authorization procedure for a berberine–silymarin combination preparation from *Berberis aristata* (Commission Implementing Decision C(2025)2494), reflecting ongoing safety uncertainty under EU novel food regulations [79,83]. EFSA’s parallel safety assessment of berberine in plant preparations, covering genotoxicity, carcinogenicity, hepatotoxicity, and drug interaction endpoints, has also progressed: the NDA Panel endorsed a draft opinion in January 2026, and the subsequent public consultation closed in May 2026 [79]. This regulatory process introduces further uncertainty for berberine-containing functional food formulations in the EU market pending the final opinion.

Urolithin A has the most advanced regulatory status. The Mitopure formulation (Amazentis SA/Timeline) received FDA GRAS designation in the United States in 2018 [84]. In the European Union, urolithin A is currently under review by EFSA as a novel food ingredient [85]. This regulatory trajectory, including the most advanced novel food application status among the four compounds, makes urolithin A the most legally straightforward ingredient for inclusion in novel functional food products in the EU.

## 9. Conclusions and Future Directions

This narrative review has examined four naturally occurring bioactive compounds, spermidine, fisetin, berberine, and urolithin A, as emerging functional food ingredients with distinct and potentially complementary effects, supported by evidence of varying clinical maturity, on key hallmarks of human aging. These compounds address non-overlapping molecular targets: autophagy induction (spermidine), senolysis (fisetin), metabolic and microbiome regulation (berberine), and selective mitophagy (urolithin A). These targets are mechanistically interconnected within the hallmarks of aging network, providing a rational basis for considering the four compounds together as a complementary multi-target dietary strategy.

The evidence base for each compound individually ranges from strong to emerging. Berberine possesses the most extensive clinical dataset, with robust meta-analytic evidence across 46 randomized controlled trials demonstrating significant and consistent reductions in glycemic, lipid, and inflammatory parameters [46]. Urolithin A has accumulated the most extensive peer-reviewed clinical dataset among naturally occurring mitophagy-inducing compounds, with completed and ongoing trials documenting safety, target engagement (mitochondrial gene expression modulation in skeletal muscle biopsy), functional improvements in muscle strength and endurance, and, most recently, immunological remodeling involving immune aging-related phenotypes [58,59,60,61,62]. Nevertheless, important translational caveats apply to the UA evidence base. Most studies have evaluated surrogate endpoints (mitochondrial gene expression, muscle biomarkers) rather than hard clinical outcomes such as falls, hospitalization, or functional independence in activities of daily living. The marked interindividual variability in UA production capacity (a substantial proportion of individuals across studied populations are suboptimal converters) further complicates interpretation of dietary intervention studies in which metabotype stratification was not applied. Spermidine has prospective epidemiological evidence from the Bruneck Study linking higher dietary intake to reduced all-cause mortality [24], while phase II and phase IIb trials have established safety but provided inconclusive evidence regarding cognitive efficacy [25,26]. Fisetin remains in the earliest clinical stage of the four compounds, with several ongoing trials and strong preclinical and ex vivo human tissue evidence for senolytic activity [29,30,32]. A comparative assessment of the current evidence base and translational readiness of the reviewed compounds is presented in Table 4.

As shown in Table 4, substantial differences exist between mechanistic promise and clinical validation. While berberine, spermidine, and urolithin A have accumulated considerable human evidence, fisetin remains at an earlier stage of clinical development. Berberine currently possesses the most extensive clinical evidence base among the reviewed compounds, although most studies have focused on metabolic rather than aging-specific outcomes. Furthermore, despite a strong mechanistic rationale for combination strategies, direct evidence supporting additional benefits from combined administration remains limited and warrants further investigation.

The multi-target hypothesis that combined administration of these four compounds could produce complementary or additive effects on aging biomarkers and functional outcomes exceeding those of individual agents is supported by mechanistic logic and network biology principles, but direct prospective evidence is currently limited to one registered clinical trial combining urolithin A and fisetin (NCT06990256) [72]. The autophagy-senescence threshold model (Bahar et al., 2026) offers a particularly compelling conceptual framework: spermidine-induced autophagy prevents senescence initiation in pre-senescent cells, while fisetin-mediated senolysis eliminates established senescent cells that have crossed the damage threshold, representing two sequential, non-redundant interventions addressing the same continuum [66]. Berberine’s microbiome reshaping reduces the gut-derived inflammatory input that accelerates both senescence and mitochondrial dysfunction, and urolithin A’s mitophagy activation addresses the organelle-level quality control impairment that characterizes senescent cells and aged tissues alike [42,57].

From a functional food science perspective, the four compounds present both opportunities and challenges. Spermidine’s presence in conventional foods (wheat germ, legumes, fermented products) at measurable concentrations enables dietary enrichment strategies without novel food authorization requirements. Fisetin’s natural occurrence in strawberries and other common fruits offers a food-compatible dietary origin, though pharmacokinetic constraints require delivery system innovation to achieve therapeutically relevant tissue exposure. Berberine’s status as a botanical medicine rather than a conventional food constituent, combined with emerging regulatory uncertainty in the European Union following the April 2025 termination of its novel food authorization procedure [83] and the ongoing EFSA safety assessment [79], means that its inclusion in EU functional food products requires careful regulatory navigation pending the outcome of these assessments. Urolithin A occupies the most favorable regulatory position, with FDA GRAS designation and an ongoing EFSA novel food application under review [84,85], making it the most legally straightforward ingredient for novel functional food formulations targeting aging-related mechanisms [86].

To translate this concept into evidence-based nutritional strategies, several key questions require systematic investigation. First, dedicated multi-compound co-administration trials in well-characterized aging populations, incorporating composite biomarker endpoints spanning autophagy, senescence, mitochondrial function, and metabolic health, are needed to test the multi-compound hypothesis prospectively. The results of NCT06990256 (urolithin A + fisetin) will provide the first human data point and should inform the design of more comprehensive multi-compound trials. Second, pharmacokinetic characterization of the four compounds in co-administration is essential, with particular attention to berberine’s established inhibitory effects on CYP3A4, CYP2D6, and P-glycoprotein, which could modulate the systemic exposure of co-administered fisetin and urolithin A [78]. Third, population stratification by urolithin metabotype (A, B, or 0) is critical for trials incorporating dietary ellagitannin strategies, as a substantial proportion of individuals lack efficient conversion capacity and would require direct urolithin A supplementation to achieve meaningful systemic exposure [51,52]. Fourth, the development of validated composite aging biomarker indices, integrating measures of autophagic flux, senescent cell burden, mitochondrial quality, and metabolic insulin sensitivity, would substantially enhance the ability to detect multi-hallmark effects in clinical trials of moderate duration and sample size.

From a practical dietary perspective, a rational multi-compound strategy need not rely exclusively on supplementation. A Mediterranean-style dietary pattern enriched in wheat germ (spermidine), strawberries and apples (fisetin), and pomegranate and walnuts (ellagitannin precursors of urolithin A), combined with berberine as a standardized botanical supplement (given its absence from conventional Western foods at pharmacologically relevant concentrations), could provide several of these compounds simultaneously within a coherent dietary framework. Such an approach aligns with the existing evidence that Mediterranean dietary adherence is associated with reduced biological aging as measured by epigenetic clocks and inflammatory biomarkers [87], and would be amenable to pragmatic clinical testing without the regulatory and formulation complexities of novel multi-compound products. Berberine would require targeted supplementation as the dietary element least achievable through food alone, and urolithin A delivery would need to account for metabotype heterogeneity as discussed above.

Functional food science is entering an era in which multi-target, mechanism-informed bioactive compound strategies, grounded in aging biology and supported by translational clinical evidence, are increasingly feasible. The four compounds differ substantially in their pharmacokinetic profiles, with important implications for formulation design and clinical translation. Their integration into evidence-based functional food products will require rigorous clinical evaluation, population-level biomarker stratification, and carefully navigated regulatory pathways. Achieving this integration represents an important, although methodologically demanding, direction for future functional food research. Overall, the available evidence supports further investigation of spermidine, fisetin, berberine, and urolithin A as potentially complementary components of functional food strategies aimed at promoting healthy aging.

## Figures and Tables

**Table 1:** Mechanistic complementarity of the four reviewed bioactive compounds within the hallmarks of aging framework.

Compound	Primary Mechanism	Aging Hallmarks Targeted	Key Signaling Nodes	Principal Source
Spermidine	General autophagy induction	Loss of proteostasis; disabled macroautophagy; chronic inflammation [2,3]	EP300 ↓; AMPK ↑; SIRT1 ↑; mTORC1 ↓ [12,13,14]	Wheat germ, soy, mushrooms, fermented foods [9,10,12]
Fisetin	Senolysis and SASP suppression	Cellular senescence; chronic inflammation; altered intercellular communication [2,3]	PI3K/AKT ↓; Bcl-2/Bcl-xL ↓; NF-κB ↓; Nrf2 ↑ [29,30,31]	Strawberries, apples, persimmons, onions [28]
Berberine	Metabolic regulation and microbiome modulation	Deregulated nutrient-sensing; dysbiosis; chronic inflammation [2,3]	AMPK ↑; mTORC1 ↓; NF-κB ↓; gut microbiota reshaping [40,69,70]	*Berberis* spp., *Coptis* spp. (nutraceutical/supplement) [39,71]
Urolithin A	Selective mitophagy induction	Mitochondrial dysfunction; chronic inflammation; immunosenescence [2,3]	PINK1/Parkin ↑; SIRT1/PGC-1α ↑; NLRP3 ↓ [54,55]	Postbiotic from ellagitannins (pomegranate, berries, walnuts) [52,53]

↑—indicates activation or upregulation; ↓—indicates inhibition or downregulation.

**Table 2:** Comparative pharmacokinetic and bioavailability parameters of the four reviewed compounds.

Compound	Oral Bioavailability	Tmax (h)	t½ (h)	Effective Human Dose	Bioavailability Strategies
Spermidine	Moderate; diet-equivalent	~1–2	Variable (see text)	0.9–3.3 mg/day (extract) [25,26]	Wheat germ extract; standardized foods [9,10]
Fisetin	Low (<10%)	~1–2	Not well characterized in humans	100–1000 mg/day (intermittent) [29,30]	Galactomannan complex; liposomes; nanoparticles [38,81]
Berberine	Very low (1–5% systemic)	~1–2	~3–5	900–1500 mg/day (divided doses) [45,46]	Dihydroberberine; phospholipid complex; SEDDS [80]
Urolithin A	Good (direct supplement); variable from diet	~4–6	~20	500–1000 mg/day	Purified supplement (Mitopure); microbiome optimization [54,82]

**Table 3:** Regulatory status of the four reviewed compounds in major jurisdictions (as of 2025/2026).

Compound	USA (FDA)	European Union (EFSA/EC)	Regulatory Concern
Spermidine	Dietary supplement; GRAS self-affirmation (synthetic) [77]	Food supplement (wheat germ extract); no novel food required [12]	None identified
Fisetin	Dietary supplement	Food supplement (plant extract); no novel food currently required	Low; advancing clinical use may trigger review
Berberine	Dietary supplement	EFSA safety assessment ongoing (2023–2026; public consultation closed May 2026); novel food authorization terminated (April 2025) [79,83]	Moderate–High; EU regulatory uncertainty pending EFSA opinion
Urolithin A	GRAS (Mitopure, 2018) [84]	Novel food application under EFSA review	Low; most advanced regulatory pathway

**Table 4:** Comparative assessment of clinical evidence and translational readiness of the reviewed compounds.

Compound	Principal Hallmark(s) Targeted	Evidence Hierarchy	Clinical Evidence	Translational Readiness	Main Limitations
Spermidine	Loss of proteostasis; impaired autophagy	In vitro + animal + observational + phase II/IIb RCT	Prospective epidemiological evidence links higher dietary intake to reduced all-cause mortality, while phase II/IIb trials support safety but have not established cognitive efficacy; cardiovascular outcomes are currently under investigation [24,25,26,27].	Moderate	Limited number of large multicenter trials and lack of long-term clinical outcome data.
Fisetin	Cellular senescence; SASP modulation	In vitro + animal + early-phase human (no completed efficacy RCT)	Clinical evidence remains limited, with most support derived from preclinical studies and ongoing clinical trials [29,30,32,33].	Low	Insufficient number of completed randomized controlled trials and limited oral bioavailability.
Berberine	Deregulated nutrient sensing; altered gut microbiome; chronic inflammation	In vitro + animal + multiple RCTs + meta-analyses	Multiple randomized controlled trials and meta-analyses demonstrate beneficial effects on glucose metabolism, lipid profiles, and inflammatory markers [46,47,48,49].	High	Most clinical studies focus on metabolic disorders rather than aging-specific endpoints.
Urolithin A	Mitochondrial dysfunction; impaired mitophagy	In vitro + animal + phase I + multiple RCTs (5 completed, *n* = 250 [58])	Completed and ongoing human clinical studies have demonstrated safety, target engagement, and improvements in mitochondrial and muscle-related outcomes [58,59,60,61,62].	Moderate–High	Long-term effects on healthy aging remain uncertain; interindividual variability may result from differences in gut microbiota composition.
Combined strategy	Multiple hallmarks of aging	Mechanistic + one registered RCT (ongoing)	Strong mechanistic rationale based on complementary biological pathways; however, direct clinical validation remains limited to early-stage investigation [66,72].	Low	Benefits beyond those of the individual compounds remain hypothetical and require experimental and clinical confirmation.

## Data Availability

No new data were created or analyzed in this study. Data sharing is not applicable to this article.

## Abbreviations

AMP	adenosine monophosphate
AMPK	AMP-activated protein kinase
ARE	antioxidant response element
ATG	autophagy-related gene/protein
ATP	adenosine triphosphate
Bcl-2	B-cell lymphoma 2
Bcl-xL	B-cell lymphoma extra-large
BMAL1	brain and muscle ARNT-like 1
cGAS	cyclic GMP-AMP synthase
CRP	C-reactive protein
CYP2D6	cytochrome P450 2D6
CYP3A4	cytochrome P450 3A4
EC	European Commission
EFSA	European Food Safety Authority
eIF5A	eukaryotic translation initiation factor 5A
EP300	E1A binding protein p300
EU	European Union
FDA	US Food and Drug Administration
FOXO	forkhead box O transcription factor
GLUT4	glucose transporter type 4
GRAS	generally recognized as safe
GSK-3β	glycogen synthase kinase 3 beta
HbA1c	glycated hemoglobin A1c
HOMA-IR	homeostatic model assessment of insulin resistance
IL-6	interleukin-6
IL-8	interleukin-8
LC3	microtubule-associated protein 1A/1B-light chain 3
LDL-C	low-density lipoprotein cholesterol
MMP	matrix metalloproteinase
mTOR	mechanistic target of rapamycin
mTORC1	mechanistic target of rapamycin complex 1
NF-κB	nuclear factor kappa-light-chain-enhancer of activated B cells
NLRP3	NOD-like receptor thermal protein domain associated protein 3
Nrf2	nuclear factor erythroid 2-related factor 2
P-gp	P-glycoprotein
PER2	period circadian regulator 2
PGC-1α	peroxisome proliferator-activated receptor gamma coactivator 1-alpha
PI3K	phosphatidylinositol 3-kinase
PINK1	PTEN-induced kinase 1
PRKAA2	protein kinase AMP-activated catalytic subunit alpha 2
RCT	randomized controlled trial
ROS	reactive oxygen species
SASP	senescence-associated secretory phenotype
SCFA	short-chain fatty acid
SEDDSs	self-emulsifying drug delivery systems
SIRT1	sirtuin 1
SREBP-1c	sterol regulatory element-binding protein 1c
STING	stimulator of interferon genes
TNF-α	tumor necrosis factor alpha
TSCM	T memory stem cell
UA	urolithin A

## References

[1] World Health Organization Ageing and Health. WHO Fact Sheet. https://www.who.int/news-room/fact-sheets/detail/ageing-and-health.

[2] López-Otín C., Blasco M.A., Partridge L., Serrano M., Kroemer G. (2013). The hallmarks of aging. Cell.

[3] López-Otín C., Blasco M.A., Partridge L., Serrano M., Kroemer G. (2023). Hallmarks of aging: An expanding universe. Cell.

[4] Aman Y., Schmauck-Medina T., Hansen M., Morimoto R.I., Simon A.K., Bjedov I., Palikaras K., Simonsen A., Johansen T., Tavernarakis N. (2021). Autophagy in healthy aging and disease. Nat. Aging.

[5] Granato D., Barba F.J., Bursać Kovačević D., Lorenzo J.M., Cruz A.G., Putnik P. (2020). Functional foods: Product development, technological trends, efficacy testing, and safety. Annu. Rev. Food Sci. Technol..

[6] Arshad Z., Shahid S., Hasnain A., Yaseen E., Rahimi M. (2025). Functional foods enriched with bioactive compounds: Therapeutic potential and technological innovations. Food Sci. Nutr..

[7] Borsky P., Holmannova D., Soukup O., Maresova T., Hanzlova M., Philipp T., Borska L. (2025). Distinct roles of urolithin A and spermidine in mitophagy and autophagy: Implications for dietary supplementation. Nutr. Res. Rev..

[8] Murillo-Cancho A.F., Martín-Latorre M.M., Lozano-Paniagua D., Nievas-Soriano B.J. (2025). Caloric restriction mimetics: Effects of spermidine and berberine on healthy longevity and prevention of aging-associated diseases. Nutr. Clín. Diet. Hosp..

[9] Muñoz-Esparza N.C., Latorre-Moratalla M.L., Comas-Basté O., Toro-Funes N., Veciana-Nogués M.T., Vidal-Carou M.C. (2019). Polyamines in food. Front. Nutr..

[10] Madeo F., Bauer M.A., Carmona-Gutierrez D., Kroemer G. (2019). Spermidine: A physiological autophagy inducer acting as an anti-aging vitamin in humans?. Autophagy.

[11] Mohajeri M., Ayatollahi S.A., Kobarfard F., Goli M., Khandan M., Mokhtari S., Khodadoost M. (2023). Wheat germ, a byproduct of the wheat milling industry, as a beneficial source of anti-aging polyamines: A quantitative comparison of various forms. Food Sci. Nutr..

[12] Madeo F., Eisenberg T., Pietrocola F., Kroemer G. (2018). Spermidine in health and disease. Science.

[13] Hofer S.J., Simon A.K., Bergmann M., Eisenberg T., Kroemer G., Madeo F. (2022). Mechanisms of spermidine-induced autophagy and geroprotection. Nat. Aging.

[14] Pietrocola F., Lachkar S., Enot D.P., Niso-Santano M., Bravo-San Pedro J.M., Sica V., Izzo V., Maiuri M.C., Madeo F., Mariño G. (2015). Spermidine induces autophagy by inhibiting the acetyltransferase EP300. Cell Death Differ..

[15] Watroba M., Szukiewicz D. (2021). Sirtuins at the service of healthy longevity. Front. Physiol..

[16] Hofer S.J., Liang Y., Zimmermann A., Schroeder S., Dengjel J., Kroemer G., Eisenberg T., Sigristd T.S., Madeo F. (2021). Spermidine-induced hypusination preserves mitochondrial and cognitive function during aging. Autophagy.

[17] Zhou J., Pang J., Tripathi M., Ho J.P., Widjaja A.A., Shekeran S.G., Cook S.A., Suzuki A., Diehl A.M., Petretto E. (2022). Spermidine-mediated hypusination of translation factor EIF5A improves mitochondrial fatty acid oxidation and prevents non-alcoholic steatohepatitis progression. Nat. Commun..

[18] Hofer S.J., Daskalaki I., Bergmann M., Friščić J., Zimmermann A., Mueller M.I., Abdellatif M., Nicastro R., Masser S., Durand S. (2024). Spermidine is essential for fasting-mediated autophagy and longevity. Nat. Cell Biol..

[19] Eisenberg T., Abdellatif M., Schroeder S., Primessnig U., Stekovic S., Pendl T., Harger A., Schipke J., Zimmermann A., Schmidt A. (2016). Cardioprotection and lifespan extension by the natural polyamine spermidine. Nat. Med..

[20] Eisenberg T., Knauer H., Schauer A., Büttner S., Ruckenstuhl C., Carmona-Gutierrez D., Ring J., Schroeder S., Magnes C., Antonacci L. (2009). Induction of autophagy by spermidine promotes longevity. Nat. Cell Biol..

[21] Ni Y.-Q., Liu Y.-S. (2021). New insights into the roles and mechanisms of spermidine in aging and age-related diseases. Aging Dis..

[22] Al-Habsi M., Chamoto K., Matsumoto K., Nomura N., Zhang B., Sugiura Y., Sonomura K., Maharani A., Nakajima Y., Wu Y. (2022). Spermidine activates mitochondrial trifunctional protein and improves antitumor immunity in mice. Science.

[23] Chamoto K., Zhang B., Tajima M., Honjo T., Fagarasan S. (2024). Spermidine—An old molecule with a new age-defying immune function. Trends Cell Biol..

[24] Kiechl S., Pechlaner R., Willeit P., Notdurfter M., Paulweber B., Willeit K., Werner P., Ruckenstuhl C., Iglseder B., Weger S. (2018). Higher spermidine intake is linked to lower mortality: A prospective population-based study. Am. J. Clin. Nutr..

[25] Schwarz C., Stekovic S., Wirth M., Benson G., Royer P., Sigrist S.J., Pieber T., Dammbrueck C., Magnes C., Eisenberg T. (2018). Safety and tolerability of spermidine supplementation in mice and older adults with subjective cognitive decline. Aging.

[26] Schwarz C., Benson G.S., Horn N., Wurdack K., Grittner U., Schilling R., Märschenz S., Köbe T., Sebastian J., Hofer S.J. (2022). Effects of spermidine supplementation on cognition and biomarkers in older adults with subjective cognitive decline: A randomized clinical trial. JAMA Netw. Open.

[27] Thorup C.V., Jeppesen C.N.A., Jensen T.H., Tinggaard A.B., Hvas C.L., Rud C.L., Skou M.K., Mortensen J.K., Reggiori F., Dengjel J. (2025). POLYamine treatment in elderly patients with Coronary Artery Disease (POLYCAD): Study protocol for a Danish randomized, double-blind, placebo-controlled trial of spermidine treatment versus placebo. Trials.

[28] Arai Y., Watanabe S., Kimira M., Shimoi K., Mochizuki R., Kinae N. (2000). Dietary intakes of flavonols, flavones and isoflavones by Japanese women and the inverse correlation between quercetin intake and plasma LDL cholesterol concentration. J. Nutr..

[29] Yousefzadeh M.J., Zhu Y., McGowan S.J., Angelini L., Fuhrmann-Stroissnigg H., Xu M., Ling Y.Y., Melos K.I., Pirtskhalava T., Inman C.L. (2018). Fisetin is a senotherapeutic that extends health and lifespan. eBioMedicine.

[30] Murray K.O., Mahoney S.A., Ludwig K.R., Miyamoto-Ditmon J.H., VanDongen N.S., Banskota N., Herman A.B., Seals D.R., Mankowski R.T., Rossman M.J. (2025). Intermittent supplementation with fisetin improves physical function and decreases cellular senescence in skeletal muscle with aging: A comparison to genetic clearance of senescent cells and synthetic senolytic approaches. Aging Cell.

[31] Tavenier J., Nehlin J.O., Houlind M.B., Rasmussen L.J., Tchkonia T., Kirkland J.L., Andersen O., Line Jee Hartmann Rasmussen L.J.H. (2024). Fisetin as a senotherapeutic agent: Evidence and perspectives. Mech. Ageing Dev..

[32] Zhu Y., Doornebal E.J., Pirtskhalava T., Giorgadze N., Wentworth M., Fuhrmann-Stroissnigg H., Laura J., Niedernhofer L.J., Paul D., Robbins P.D. (2017). New agents that target senescent cells: The flavone, fisetin, and the BCL-XL inhibitors, A1331852 and A1155463. Aging.

[33] Kim S., Choi K.J., Cho S.-J., Yun S.-M., Jeon J.-P., Koh Y.-H., Song J., Johnson G.V.W., Jo C. (2016). Fisetin stimulates autophagic degradation of phosphorylated tau via the activation of TFEB and Nrf2 transcription factors. Sci. Rep..

[34] Currais A., Prior M., Dargusch R., Armando A., Ehren J., Schubert D., Quehenberger O., Maher P. (2014). Modulation of p25 and inflammatory pathways by fisetin maintains cognitive function in Alzheimer’s disease transgenic mice. Aging Cell.

[35] Maher P. (2024). The flavonoid fisetin reduces multiple physiological risk factors for dementia. Neurochem. Int..

[36] Khan S., Khan M.M., Badruddeen, Ahmad U., Khan S. (2025). Fisetin as a multifunctional bioactive flavonoid: A comprehensive review of its pharmacology, mechanism of action, safety profile, clinical trials, and future directions in therapeutics. Food Biosci..

[37] Silva M., Wacker D.A., Driver B.E., Staugaitis A., Niedernhofer L.J., Schmidt E.L., Kirkland J.L., Tchkonia T., Evans T., Serrano C.H. (2024). Senolytics to slow progression of sepsis (STOP-Sepsis) in elderly patients: Study protocol for a multicenter, randomized, adaptive allocation clinical trial. Trials.

[38] Krishnakumar I.M., Jaja-Chimedza A., Joseph A., Balakrishnan A., Maliakel B., Swick A. (2022). Enhanced bioavailability and pharmacokinetics of a novel hybrid-hydrogel formulation of fisetin orally administered in healthy individuals: A randomized double-blinded comparative crossover study. J. Nutr. Sci..

[39] Neag M.A., Mocan A., Echeverría J., Pop R.M., Bocsan C.I., Crişan G., Buzoianu A.D. (2018). Berberine: Botanical occurrence, traditional uses, extraction methods, and relevance in cardiovascular, metabolic, hepatic, and renal disorders. Front. Pharmacol..

[40] Mbara K.C., Kheoane P.S., Tarirai C. (2025). Targeting AMPK signaling: The therapeutic potential of berberine in diabetes and its complications. Pharmacol. Res. Mod. Chin. Med..

[41] Wang P., Li R., Li Y., Tan S., Jiang J., Liu H., Wei X. (2023). Berberine alleviates non-alcoholic hepatic steatosis partially by promoting SIRT1 deacetylation of CPT1A in mice. Gastroenterol. Rep..

[42] Yu Y., Zhao Y., Teng F., Li J., Guan Y., Xu J., Lv X., Guan F., Zhang M., Chen L. (2018). Berberine improves cognitive deficiency and muscular dysfunction via activation of the AMPK/SIRT1/PGC-1α pathway in skeletal muscle from naturally aging rats. J. Nutr. Health Aging.

[43] He Q., Dong H., Guo Y., Gong M., Xia Q., Lu F., Wang D. (2022). Multi-target regulation of intestinal microbiota by berberine to improve type 2 diabetes mellitus. Front. Endocrinol..

[44] Bei Y., Wang T., Guan S. (2025). Berberine extends lifespan in *C. elegans* through multi-target synergistic antioxidant effects. Antioxidants.

[45] Yin J., Xing H., Ye J. (2008). Efficacy of berberine in patients with type 2 diabetes mellitus. Metabolism.

[46] Guo J., Chen H., Zhang X., Lou W., Zhang P., Qiu Y., Zhang C., Wang Y., Liu W.J. (2021). The effect of berberine on metabolic profiles in type 2 diabetic patients: A systematic review and meta-analysis of randomized controlled trials. Oxidative Med. Cell. Longev..

[47] Nazari A., Ghotbabadi Z.R., Kazemi K.S., Metghalchi Y., Tavakoli R., Rahimabadi R.Z., Ghaheri M. (2024). The effect of berberine supplementation on glycemic control and inflammatory biomarkers in metabolic disorders: An umbrella meta-analysis of randomized controlled trials. Clin. Ther..

[48] Zhang Y., Gu Y., Ren H., Wang S., Zhong H., Zhao X., Ma J., Gu X., Xue Y., Huang S. (2020). Gut microbiome-related effects of berberine and probiotics on type 2 diabetes (the PREMOTE study). Nat. Commun..

[49] Harrison S.A., Gunn N., Neff G.W., Kohli A., Liu L., Flyer A., Lawrence Goldkind L., Di Bisceglie A.M. (2021). A phase 2, proof of concept, randomized controlled trial of berberine ursodeoxycholate in patients with presumed non-alcoholic steatohepatitis and type 2 diabetes. Nat. Commun..

[50] Hua Z., Wu Q., Yang Y., Liu S., Jennifer T.G., Zhao D., Fang Y. (2024). Essential roles of ellagic acid-to-urolithin converting bacteria in human health and health food industry: An updated review. Trends Food Sci. Technol..

[51] He F., Bian Y., Zhao Y., Xia M., Liu S., Gui J., Hou X., Fang Y. (2024). In vitro conversion of ellagic acid to urolithin A by different gut microbiota of urolithin metabotype A. Appl. Microbiol. Biotechnol..

[52] Iglesias-Aguirre C.E., García-Villalba R., Beltrán D., Frutos-Lisón M.D., Espín J.C., Tomás-Barberán F.A., Selma M.V. (2023). Gut bacteria involved in ellagic acid metabolism to yield human urolithin metabotypes revealed. J. Agric. Food Chem..

[53] García-Villalba R., Giménez-Bastida J.A., Cortés-Martín A., Ávila-Gálvez M.Á., Tomás-Barberán F.A., Selma M.V., Espín J.C., González-Sarrías A. (2022). Urolithins: A comprehensive update on their metabolism, bioactivity, and associated gut microbiota. Mol. Nutr. Food Res..

[54] Ryu D., Mouchiroud L., Andreux P.A., Katsyuba E., Moullan N., Nicolet-dit-Félix A.A., Williams E.G., Jha P., Lo Sasso G., Huzard D. (2016). Urolithin A induces mitophagy and prolongs lifespan in *C. elegans* and increases muscle function in rodents. Nat. Med..

[55] Narendra D.P., Youle R.J. (2024). The role of PINK1–Parkin in mitochondrial quality control. Nat. Cell Biol..

[56] Luan P., D’Amico D., Andreux P.A., Laurila P.-P., Wohlwend M., Li H., de Lima T.I., Place N., Rinsch C., Zanou N. (2021). Urolithin A improves muscle function by inducing mitophagy in muscular dystrophy. Sci. Transl. Med..

[57] Liu S., Faitg J., Tissot C., Konstantopoulos D., Laws R., Bourdier G., Andreux P.A., Davey T., Gallart-Ayala H., Ivanisevic J. (2025). Urolithin A provides cardioprotection and mitochondrial quality enhancement preclinically and improves human cardiovascular health biomarkers. iScience.

[58] Kuerec A.H., Lim X.K., Khoo A.L.Y., Sandalova E., Guan L., Feng L., Maier A.B. (2024). Targeting aging with urolithin A in humans: A systematic review. Ageing Res. Rev..

[59] Andreux P.A., Blanco-Bose W., Ryu D., Burdet F., Ibberson M., Aebischer P., Johan Auwerx J., Singh A., Rinsch C. (2019). The mitophagy activator urolithin A is safe and induces a molecular signature of improved mitochondrial and cellular health in humans. Nat. Metab..

[60] Singh A., D’Amico D., Andreux P.A., Fouassier A.M., Blanco-Bose W., Evans M., Aebischer P., ∙ Johan Auwerx J., Rinsch C. (2022). Urolithin A improves muscle strength, exercise performance, and biomarkers of mitochondrial health in a randomized trial in middle-aged adults. Cell Rep. Med..

[61] Liu S., D’Amico D., Shankland E., Bhayana S., Garcia J.M., Aebischer P., Rinsch C., Singh A., Marcinek D.J. (2022). Effect of urolithin A supplementation on muscle endurance and mitochondrial health in older adults: A randomized clinical trial. JAMA Netw. Open.

[62] Denk D., Singh A., Kasler H.G., D’Amico D., Rey J., Alcober-Boquet L., Gorol J.M., Steup C., Tiwari R., Kwok R. (2025). Effect of the mitophagy inducer urolithin A on age-related immune decline: A randomized, placebo-controlled trial. Nat. Aging.

[63] Jiménez-Loygorri J.I., Villarejo-Zori B., Viedma-Poyatos Á., Zapata-Muñoz J., Benítez-Fernández R., Frutos-Lisón M.D., Tomás-Barberán F.A., Espín J.C., Area-Gómez E., Gomez-Duran A. (2024). Mitophagy curtails cytosolic mtDNA-dependent activation of cGAS/STING inflammation during aging. Nat. Commun..

[64] Swanson K.V., Deng M., Ting J.P.-Y. (2019). The NLRP3 inflammasome: Molecular activation and regulation to therapeutics. Nat. Rev. Immunol..

[65] Gulen M.F., Samson N., Keller A., Schwabenland M., Liu C., Glück S., Vivek V., Thacker V.V., Favre L., Mangeat B. (2023). cGAS–STING drives ageing-related inflammation and neurodegeneration. Nature.

[66] Bahar M.E., Hwang J.S., Lai T.H., Akter K.-M., Maulidi R.F., Kim D.R. (2026). The autophagy-senescence axis as a threshold model of aging and therapeutic targeting. Redox Biol..

[67] Guilbaud E., Sarosiek K.A., Galluzzi L. (2024). Inflammation and mitophagy are mitochondrial checkpoints to aging. Nat. Commun..

[68] Fernandes S.A., Demetriades C. (2021). The multifaceted role of nutrient sensing and mTORC1 signaling in physiology and aging. Front. Aging.

[69] Meyers A.K., Zhu X. (2020). The NLRP3 inflammasome: Metabolic regulation and contribution to inflammaging. Cells.

[70] Yang F., Gao R., Luo X., Liu R., Xiong D. (2023). Berberine influences multiple diseases by modifying gut microbiota. Front. Nutr..

[71] Nadalin P., Kim Y.-G., Park S.U. (2023). Recent studies on berberine and its biological and pharmacological activities. EXCLI J..

[72] (2025). Evaluation of Urolithin A and Fisetin on Improving Sleep and Aging Biomarkers in Middle-Aged and Older Adults. Huazhong University of Science and Technology/Bonerge. ClinicalTrials.gov Identifier NCT06990256. NCT06990256.

[73] Baker D.J., Wijshake T., Tchkonia T., LeBrasseur N.K., Childs B.G., van de Sluis B., Kirkland J.L., van Deursen J.M. (2011). Clearance of p16Ink4a-positive senescent cells delays ageing-associated disorders. Nature.

[74] Baker D.J., Childs B.G., Durik M., Wijers M.E., Sieben C.J., Zhong J., Saltness R.A., Jeganathan K.B., Verzosa G.C., Pezeshki A. (2016). Naturally occurring p16Ink4a-positive cells shorten healthy lifespan. Nature.

[75] Victorelli S., Salmonowicz H., Chapman J., Martini H., Vizioli M.G., Riley J.S., Cloix C., Hall-Younger E., Espindola-Netto J.M., Jurk D. (2023). Apoptotic stress causes mtDNA release during senescence and drives the SASP. Nature.

[76] Petrović J., Pešić V., Lauschke V.M. (2020). Frequencies of clinically important CYP2C19 and CYP2D6 alleles are graded across Europe. Eur. J. Hum. Genet..

[77] Chrysostomou P.P., Freeman E.L., Murphy M.M., Pereira R., Esdaile D.J., Keohane P. (2024). A toxicological assessment of spermidine trihydrochloride produced using an engineered strain of *Saccharomyces cerevisiae*. Food Chem. Toxicol..

[78] Colombo D., Lunardon L., Bellia G. (2014). Cyclosporine and herbal supplement interactions. J. Toxicol..

[79] European Food Safety Authority (EFSA) Call for Data for the Scientific Opinion on the Evaluation of the Safety in Use of Plant Preparations Containing Berberine (EFSA-Q-2022-00803). EFSA, 2023; Draft Opinion Endorsed by the NDA Panel, January 2026. https://www.efsa.europa.eu/en/call/call-data-scientific-opinion-evaluation-safety-use-plant-preparations-containing-berberine.

[80] Feng R., Shou J.-W., Zhao Z.-X., He C.-Y., Ma C., Huang M., Fu J., Tan X.-S., Li X.-Y., Wen B.-Y. (2015). Transforming berberine into its intestine-absorbable form by the gut microbiota. Sci. Rep..

[81] Szymczak J., Cielecka-Piontek J. (2023). Fisetin—In search of better bioavailability—From macro to nano modifications: A review. Int. J. Mol. Sci..

[82] Faitg J., D’Amico D., Rinsch C., Singh A. (2024). Mitophagy activation by urolithin A to target muscle aging. Calcif. Tissue Int..

[83] European Commission (2025). Commission Implementing Decision (EU) C(2025)2494 of 29 April 2025 Terminating the Procedure for Authorising the Placing on the Market of a Mixture of Berberine Hydrochloride Purified from Berberis Aristata DC and Silymarin as a Novel Food. Official Journal of European Union. https://food.ec.europa.eu/food-safety/novel-food/decisions-terminating-procedure_en.

[84] U.S. Food and Drug Administration Agency Response Letter, GRAS Notice No. GRN 000791 (Urolithin A; Amazentis SA), Filed 9 July 2018. https://www.fda.gov/media/120300/download.

[85] European Commission Amazentis SA: Application for the Approval of Urolithin A as a Novel Food Under Regulation (EU) 2015/2283 (Application 2018/0538; Status: Ongoing). https://food.ec.europa.eu/document/download/8f10d273-65cb-4450-85cb-4bf389d8670c_en?filename=novel-food_sum_ongoing-app_2018-0538.pdf.

[86] Vettorazzi A., López de Cerain A., Sanz-Serrano J., Gil A.G., Azqueta A. (2020). European regulatory framework and safety assessment of food-related bioactive compounds. Nutrients.

[87] Gensous N., Garagnani P., Santoro A., Giuliani C., Ostan R., Fabbri C., Milazzo M., Gentilini D., di Blasio A.M., Pietruszka B. (2020). One-year Mediterranean diet promotes epigenetic rejuvenation with country- and sex-specific effects: A pilot study from the NU-AGE project. GeroScience.

